# Increase in the Population of Patients with Neovascular Age-Related Macular Degeneration Who Underwent Long-Term Active Treatment

**DOI:** 10.1038/s41598-019-49749-y

**Published:** 2019-09-13

**Authors:** Seung Kook Baek, Jae Hui Kim, Jong Woo Kim, Chul Gu Kim

**Affiliations:** 1 Department of Ophthalmology, Konyang University College of Medicine, Daejeon, South Korea; 2 Department of Ophthalmology, Kim’s Eye Hospital, Konyang University College of Medicine, Seoul, South Korea

**Keywords:** Drug therapy, Retinal diseases, Epidemiology

## Abstract

To investigate changes in the size of the population of patients who are receiving long-term, active treatment for neovascular age-related macular degeneration (AMD). This retrospective, observational study included 3,380 patients who received anti-vascular endothelial growth factor injections (3,974 eyes). The injections performed were divided into the following three groups: group 1, injections performed right after the initial diagnosis; group 2, injections performed <24 months; and group 3, injection performed ≥24 months. Time-dependent changes in the proportion of injections in each group were analyzed. The total number of injections markedly increased from 431 in the 1st quarter of 2014 to 1,323 in the 4th quarter of 2018. There were significant changes in the proportion of injections in each group over time (P < 0.001). The proportions of group 1, group 2, and group 3 in the 1st quarter of 2014 were 17.4%, 65.4%, and 17.2%, respectively. The proportions changed to 10.6%, 50.2%, and 39.5% in the 4th quarter of 2018, respectively. The marked increase in the proportions of group 3 may suggest an increase in the patient population that underwent long-term active treatment. The socioeconomic influence of this trend should be considered when establishing future strategies for neovascular AMD.

## Introduction

Age-related macular degeneration (AMD) is one of the primary global causes of vision impairment^1^. With the aging of global population, AMD is emerging as a very important disease, and the increasing socioeconomic burden of AMD has been raised as an important issue in global health^2–4^.

Among various presentations of AMD, neovascular AMD requires active treatment to prevent blindness^5^. However, anti-vascular endothelial growth factor (VEGF) therapy, a gold-standard treatment, is usually expensive. Additionally, frequent hospital visits are required to preserve vision. For this reason, long-term treatment of neovascular AMD may accompany an increase in economic and time constraints^6,7^. Severe deterioration of vision to the point where further treatment leads to insignificant outcomes is a disastrous outcome for the patient. However, the patients can be followed up less frequently without additional injections, suggesting reduced treatment burden to the patient, caregivers, and society.

Considering these factors, patients receiving long-term active treatment may have a much greater impact on the treatment burden of neovascular AMD than do those who are not receiving treatment. Therefore, information on the size of this patients’ population and how the size changes over time may provide important information in establishing future strategies for the treatment of neovascular AMD. To the best of our knowledge, no previous studies have focused on this subject.

The present study aimed to investigate changes in the size of the population of patients who were diagnosed with neovascular AMD and are receiving long-term active anti-VEGF treatment. We additionally discuss the potential reasons and the significance of these changes.

## Materials and Methods

This retrospective, observational study was conducted at a single center (Kim’s Eye Hospital, Seoul, South Korea). The study was approved by the Institutional Review Board of Kim’s Eye Hospital and was conducted in accordance with the tenets of the Declaration of Helsinki. The need for informed consent was waived by Institutional Review Board.

### Patients

We secured the list of patients who received anti-VEGF injection in our institution between January 1, 2014 and December 31, 2018. Among them, medical records of patients who registered to the “Registration Application for the Exempted Calculation of Health Insurance” and who received ranibizumab or aflibercept injection as an initial treatment were reviewed. In the Korean national insurance system, neovascular AMD is considered as an “intractable disorder”; hence, the Korean national insurance system implements a special medical expense support system. The national health insurance covers 90% of the expenses of ranibizumab or aflibercept treatments for the number of times as specified in the regulation. Although this system does not support the expense for bevacizumab treatment, almost of all the patients who are newly diagnosed with neovascular AMD are registered to this system.

### Outcome measure

The total number of anti-VEGF injections, including ranibizumab, aflibercept, and bevacizumab, performed between January 1, 2014 and December 31, 2018, were recorded. Then, the number of injections performed in each quarter was counted. The primary analysis was performed based on the number of the quarterly injections. The injections were divided into three groups, according to the following criteria.

**Group 1**. Injections performed right after the diagnosis of neovascular AMD: The number of injections in this group is exactly the same as the number of patients who are newly diagnosed with neovascular AMD.

**Group 2**. Injections performed <24 months after the diagnosis, excluding the first injection.

**Group 3**. Injections performed ≥24 months after the diagnosis: This suggests that the patients are still receiving active treatment even 24 months after the diagnosis, suggesting that the value reflects the size of the population of patients who underwent long-term treatment.

The time-dependent changes in the proportion of injections in each group among the total injections were estimated. In addition, the time-dependent changes in the interval between the diagnosis of neovascular AMD and the last anti-VEGF injection were calculated. If both eyes are diagnosed with neovascular AMD simultaneously or the neovascularization developed in the initially uninvolved eye during the follow-up period, all these cases are considered to have a new diagnosis of neovascular AMD and are thus analyzed separately.

Patients who registered with the “Registration Application for the Exempted Calculation of Health Insurance” during the study period but received bevacizumab injection as an initial treatment, were included in the bevacizumab group. In this group, the time-dependent changes in the interval between the diagnosis of neovascular AMD and the last anti-VEGF injection were calculated.

### Statistical analysis

Data are presented as mean ± standard deviation or number (%) where applicable. Statistical analyses were performed using a commercially available software package (Statistical Package for the Social Sciences for Windows, version 21.0; IBM, Armonk, NY, USA). Time-dependent changes in the proportion of injections in each group among the total injections were analyzed using the Friedman test. Time-dependent changes in the interval between the diagnosis of neovascular AMD and the last anti-VEGF injection were analyzed using Spearman’s correlation analysis. A P*-*value < 0.05 was considered to be statistically significant.

### Meeting presentation

Part of this study was presented at the Korean Retina Society Winter Symposium, Incheon, South Korea (February 23, 2019).

## Results

During the study period, a total of 19,262 anti-VEGF injections were performed on 4,412 eyes. Among them, 3,974 eyes (90.1%) of the 3,380 patients were initially treated with ranibizumab or aflibercept. Characteristics of these patients are summarized in Table 1. The mean age of the patient was 71.1 ± 8.5 years, and the mean duration between the diagnosis of neovascular AMD and the last injection was 18.2 ± 18.3 months.

**Table 1:** Characteristics of the 3,380 patients (3,974 eyes) who were initially treated with ranibizumab or aflibercept.

Characteristic	
Age, years	71.1 ± 8.5
50–59, No.	352 (9.6%)
60–69, No.	993 (29.4%)
70–79, No.	1,481 (43.8%)
80–, No.	544 (16.1%)
**Sex**	
Men	2,302 (68.1%)
Women	1,078 (31.9%)
**Laterality**	
Right	1,416 (41.9%)
Left	1,370 (40.5%)
Both	594 (17.6%)
Total number of anti-VEGF injections	18,165
**No. of anti-VEGF agents used**	
Ranibizumab	7,660
Aflibercept	8,546
Bevacizumab	1,959
Duration between the diagnosis of neovascular AMD and the last injection, months	18.2 ± 18.3

The data are presented as mean ± standard deviation or number (%) when applicable.

Abbreviations: AMD = age-related macular degeneration, VEGF = vascular endothelial growth factor

**Figure 1:**
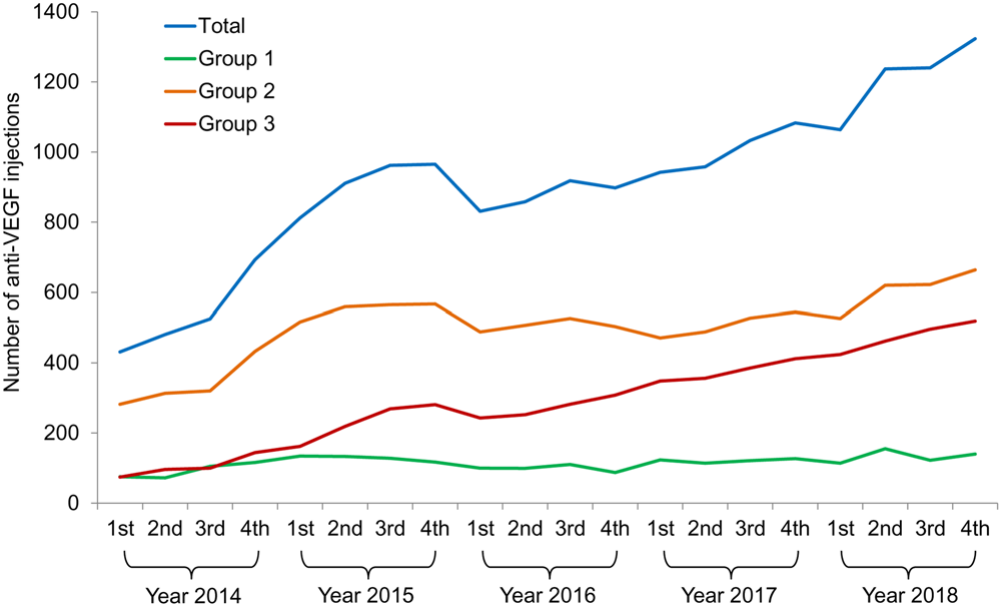
Changes in the number of anti-vascular endothelial growth factor (VEGF) injections over time, when divided into groups, according to the duration between the diagnosis of neovascular age-related macular degeneration and the injection. 1st = 1st quarter, 2nd = 2nd quarter, 3rd = 3rd quarter, 4th = 4th quarter, group 1, injections performed right after the initial diagnosis of neovascular age-related macular degeneration; group 2, injections performed <24 months after the initial diagnosis, excluding the first injection; group 3, injection performed ≥24 months after the diagnosis. Data were acquired from patients whose eyes were initially treated with ranibizumab or aflibercept.

**Figure 2:**
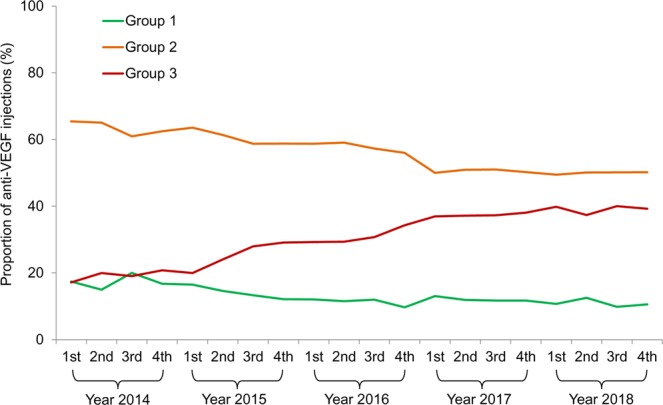
Changes in the proportion of anti-vascular endothelial growth factor (VEGF) injections over time, when divided into groups according to the duration between the diagnosis of neovascular age-related macular degeneration and the injection. 1st = 1st quarter, 2nd = 2nd quarter, 3rd = 3rd quarter, 4th = 4th quarter, group 1, injections performed right after the initial diagnosis of neovascular age-related macular degeneration; group 2, injections performed <24 months after the initial diagnosis, excluding the first injection; group 3, injection performed ≥24 months after the diagnosis. Data were acquired from patients whose eyes were initially treated with ranibizumab or aflibercept.

Table 2 shows the changes in the number of injections and proportion of injections in each group (groups 1, 2, and 3). The number of injections markedly increased from 431 injections in the 1st quarter of 2014 to 1,323 injections in the 4th quarter of 2018 (Fig. 1 and Table 2). The number of injections within the three groups also shows an increasing trend over time. Additionally, there was a significant difference in the proportion of injections in each group over time (P < 0.001, Fig. 2 and Table 2). More specifically, the proportion of injections in group 3 (injection performed ≥24 months after diagnosis of neovascular AMD) markedly increased from 17.2% in the 1st quarter of 2014 to 39.2% in the 4th quarter of 2018. In contrast, there was a decreasing trend of the proportion of injections in groups 1 and 2 over time.

**Table 2:** Total number of anti-vascular endothelial growth factor injections and proportion of injections in three groups according to the duration between the diagnosis of neovascular age-related macular degeneration (AMD) and the injection.

Years	Groups						Total (%)
	Group 1		Group 2		Group 3		
	No.	%	No.	%	No.	%	
**2014** 1st	75	17.4	282	65.4	74	17.2	431 (100)
2nd	72	14.9	313	65.1	96	19.9	481 (100)
3rd	105	20.0	320	60.9	100	19.1	525 (100)
4th	116	16.7	433	62.5	144	20.8	693 (100)
**2015** 1st	134	16.5	516	63.6	162	19.9	812 (100)
2nd	133	14.6	559	61.4	219	24.0	911 (100)
3rd	128	13.3	565	58.7	269	27.9	962 (100)
4th	117	12.1	567	58.8	281	29.1	965 (100)
**2016** 1st	100	12.0	488	58.7	243	29.2	831 (100)
2nd	99	11.5	507	59.1	252	29.4	858 (100)
3rd	110	11.9	526	57.3	282	30.7	918 (100)
4th	87	9.7	503	56.0	308	34.3	898 (100)
**2017** 1st	123	13.1	471	50.0	348	36.9	942 (100)
2nd	114	11.9	488	50.9	356	37.2	958 (100)
3rd	121	11.7	527	51.0	385	37.3	1033 (100)
4th	127	11.7	544	50.2	412	38.0	1083 (100)
**2018** 1st	114	10.7	526	49.4	424	39.9	1064 (100)
2^nd^	155	12.5	620	50.1	462	37.4	1237 (100)
3^rd^	122	9.8	622	50.2	496	40.0	1240 (100)
4^th^	140	10.6	664	50.2	519	39.2	1323 (100)

1st = 1st quarter, 2nd = 2nd quarter, 3rd = 3rd quarter, 4th = 4th quarter.

Group 1, injections performed right after the initial diagnosis of neovascular age-related macular degeneration; group 2, injections performed <24 months after the initial diagnosis, excluding the first injection; group 3, injection performed ≥24 months after the diagnosis.

**Figure 3:**
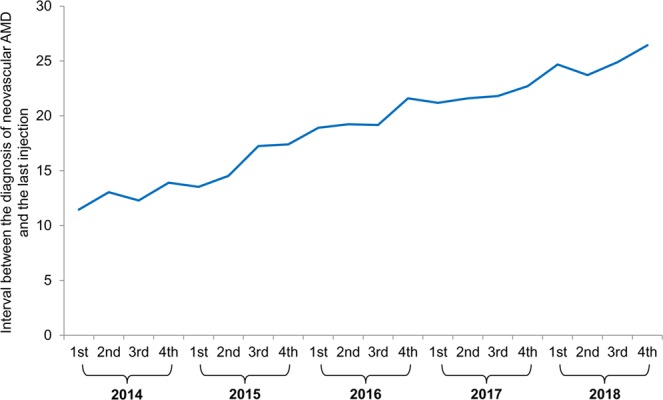
Changes in the interval between the diagnosis of neovascular age-related macular degeneration (AMD) and the last anti-vascular endothelial growth factor injection. Data were acquired from patients whose eyes were initially treated with ranibizumab or aflibercept. 1st = 1st quarter; 2nd = 2nd quarter; 3rd = 3rd quarter; 4th = 4th quarter.

Figure 3 and Table 3 shows the time-dependent changes in the interval between the diagnosis of neovascular AMD and the last anti-VEGF injection. There was a tendency of the interval to significantly increase over time (P < 0.001, r = 0.991).

**Table 3:** Mean (±standard deviation) interval between the diagnosis of neovascular age-related macular degeneration (AMD) and the last anti-vascular endothelial growth factor (VEGF) injection in patients whose eyes were initially treated with ranibizumab or aflibercept.

Years	Interval between the diagnosis of neovascular AMD and the last anti-VEGF injection	No. of eyes
**2014** 1st	11.45 ± 15.91	431
2nd	13.04 ± 16.80	481
3rd	12.28 ± 16.97	525
4th	13.90 ± 19.23	693
**2015** 1st	13.53 ± 18.64	812
2nd	14.53 ± 19.72	911
3rd	17.24 ± 21.41	962
4th	17.40 ± 21.49	965
**2016** 1st	18.91 ± 22.56	831
2nd	19.23 ± 22.53	858
3rd	19.16 ± 22.32	918
4th	21.60 ± 24.00	898
**2017** 1st	21.18 ± 23.99	942
2nd	21.60 ± 24.77	958
3rd	21.81 ± 24.84	1033
4th	22.71 ± 26.18	1083
**2018** 1st	24.68 ± 27.78	1064
2^nd^	23.71 ± 27.26	1237
3^rd^	24.89 ± 27.52	1240
4^th^	26.43 ± 30.71	1323

1st = 1st quarter, 2nd = 2nd quarter, 3rd = 3rd quarter, 4th = 4th quarter.

Four-hundred and thirty-eight eyes (9.9%) of 389 patients were initially treated with bevacizumab and were included in the bevacizumab group. Patient characteristics of the bevacizumab group are summarized in Table 4. Time-dependent changes in the number of injections are shown in Table 5. Changes in the interval between the diagnosis of neovascular AMD and the last injection are additionally presented in Table 5. There was a tendency of the interval to significantly increase over time (P < 0.001, r = 0.834).

**Table 4:** Characteristics of the 389 patients (438 eyes) who were initially treated with bevacizumab.

Characteristic	
Age, years	68.04 ± 10.12
50–59, No.	77 (19.79%)
60–69, No.	126 (32.39%)
70–79, No.	130 (33.42%)
80-, No.	56 (14.40%)
**Sex**	
Men	229 (58.87%)
Women	160 (41.13%)
**Laterality**	
Right	184 (47.30%)
Left	156 (40.10%)
Both	49 (12.60%)
Total number of anti-VEGF injections	1,097
Duration between the diagnosis of neovascular AMD and the last injection, months	4.75 ± 7.58

The data are presented as mean ± standard deviation or number (%) when applicable.

Abbreviations: AMD = age-related macular degeneration, VEGF = vascular endothelial growth factor.

**Table 5:** Mean (±standard deviation) interval between the diagnosis of neovascular age-related macular degeneration (AMD) and the last injection in patients whose eyes were initially treated with bevacizumab.

Years	Interval between the diagnosis of neovascular AMD and the last injection	No. of eyes
**2014** 1st	2.06 ± 3.03	20
2nd	3.15 ± 3.20	20
3rd	3.35 ± 3.13	14
4th	2.73 ± 2.63	13
**2015** 1st	2.73 ± 2.49	12
2nd	2.04 ± 2.73	27
3rd	2.86 ± 3.66	42
4th	2.42 ± 3.44	45
**2016** 1st	2.12 ± 3.44	49
2nd	2.83 ± 4.67	69
3rd	3.94 ± 5.82	64
4th	5.26 ± 6.95	61
**2017** 1st	3.61 ± 5.50	90
2nd	4.38 ± 6.73	89
3rd	3.94 ± 6.32	108
4th	5.04 ± 8.46	73
**2018** 1st	6.84 ± 9.93	73
2^nd^	6.08 ± 8.97	80
3^rd^	6.91 ± 9.74	76
4^th^	9.03 ± 11.22	72

1st = 1st quarter, 2nd = 2nd quarter, 3rd = 3rd quarter, 4th = 4th quarter.

## Discussion

The primary findings of the present study are as follows. First, a total number of anti-VEGF injections for neovascular AMD continuously increased over time. Second, the number of patients who are newly diagnosed with neovascular AMD also increased, but only slightly, over time. Third, the number of injections performed for patients who underwent ≥24 months of long-term active anti-VEGF treatment markedly increased over time. As a result, the proportion of these injections to the total injections in patients receiving long-term treatment was dramatically increased from 17.2% to 39.2% during the 5-year study period. Interval between the diagnosis of neovascular AMD and the last anti-VEGF injection has also increased over time. This result suggests that the increase in the number of patients undergoing long-term treatment is the primary contributor to the overall increase in the number of entire injections. The former two findings are somewhat predictable. In fact, these are similar to the recent studies showing increase in AMD patients and ranibizumab/aflibercept administration in South Korea^8,9^. However, the third finding is a new trend that according to the authors’ knowledge has not been reported in the English literature. It would be of great value to address the following questions: “Why did this trend occur?” “Will this trend continue in the future?” “How will this trend be taken into account when establishing future strategies for the treatment neovascular AMD?”

### Why did this trend occur

Severe visual deterioration where further treatment is not beneficial is one of the primary causes of treatment discontinuation in neovascular AMD^10,11^. Generally, in the case of a patient who visits a hospital continuously and receives active treatment, it can be assumed that the loss of vision is not complete and that the remaining vision needs to be maintained throughout the course of the treatment. Thus, as shown in the present study, increase in the number of patients receiving long-term active treatment may suggest that the number of patients without complete loss of vision is increasing. We cautiously postulate that this tendency is primarily caused by improved treatment outcomes due to the long-term, gradual changes in the method of anti-VEGF treatment.

With the introduction of anti-VEGF therapy, the treatment outcomes of neovascular AMD have been markedly improved. In early clinical trials, monthly fixed dosing of ranibizumab for 2 years prevented vision loss and improved mean visual acuity^5^. The more recently introduced anti-VEGF agent aflibercept also showed long-term efficacy comparable to that of ranibizumab^12^. Despite this remarkable success, the two major huddles in the application of anti-VEGF therapy in the real-world practice were high cost of the drug and the need for frequent hospital visits and injections. Since the monthly injection regimen used in clinical trials was associated with great treatment burden, investigators had focused on the development of more efficient treatment regimens. As-needed regimen^13^ was probably the first widely adopted regimen to decrease the frequency of injection. The key point of as-needed regimen is that after initial treatment, re-treatment is performed only when the fluid accumulation persists or recurs. Treat-and-extend regimen^14,15^ is another widely used treatment regimen. In this regimen, continuous injections are performed on every follow-up visit, regardless of the fluid status. The difference with fixed-dosing regimen is that the follow-up and injection interval is adjusted based on the macular finding; if there is a fluid, the interval is shortened, whereas the interval is extended if macula remains dry. In clinical practice, the 3 regimens, fixed dosing, as-needed, and treat-and-extend, are considered the basic methods in treating neovascular AMD.

More than 12 years have passed since the introduction of anti-VEGF therapy. During that period, each physician has established his/her own treatment method for the patient, through the experiences of success and failure in treating the disease and following the lessons from clinical studies. During this period, a number of studies showed two important facts. First, starting from mid-2010, studies based on large data from real-world setting have shown that long-term prognosis of neovascular AMD is relatively unfavorable compared with that reported in clinical trials^16,17^ visual improvement is noted only during the early period of treatment, and slow but continuous deterioration in visual acuity is noted thereafter. In particular, less frequent monitoring and injection were found to be the primary factors that contributed to this trend^18^. Second, the excellent efficacy of treat-and-extend regimen has been proven. Previous studies have shown that the outcome of treat-and-extend regimen is generally comparable to that of the fixed-dosing regimen^19^ and even better than that of the as-needed regimen^20,21^. Moreover, changing from the as-needed to treat-and-extend regimen is also found to be effective in improving and stabilizing patient outcomes^22,23^.

An important point revealed by these two findings was that more active, frequent anti-VEGF injections can improve treatment outcomes. As awareness regarding this aspect is increasing, it is presumed that they have influenced treatment patterns in clinical practice. In our institution, the concept of proactive treatment, such as treat-and-extend regimen, was first introduced in 2015 as a result of these consequences. Fortunately, changes in national insurance policy in South Korea also supported this trend. In South Korea, 90% of drug expenses of ranibizumab and aflibercept for neovascular AMD are covered by national health insurance. However, there has been a limit on the number of times these benefits can be given. During the recent years, the number of insurance-covered injections has been increased, reducing the economic burden of patients undergoing multiple injections^9^. This change is thought to have become one of the bases for more active treatment.

**Figure 4:**
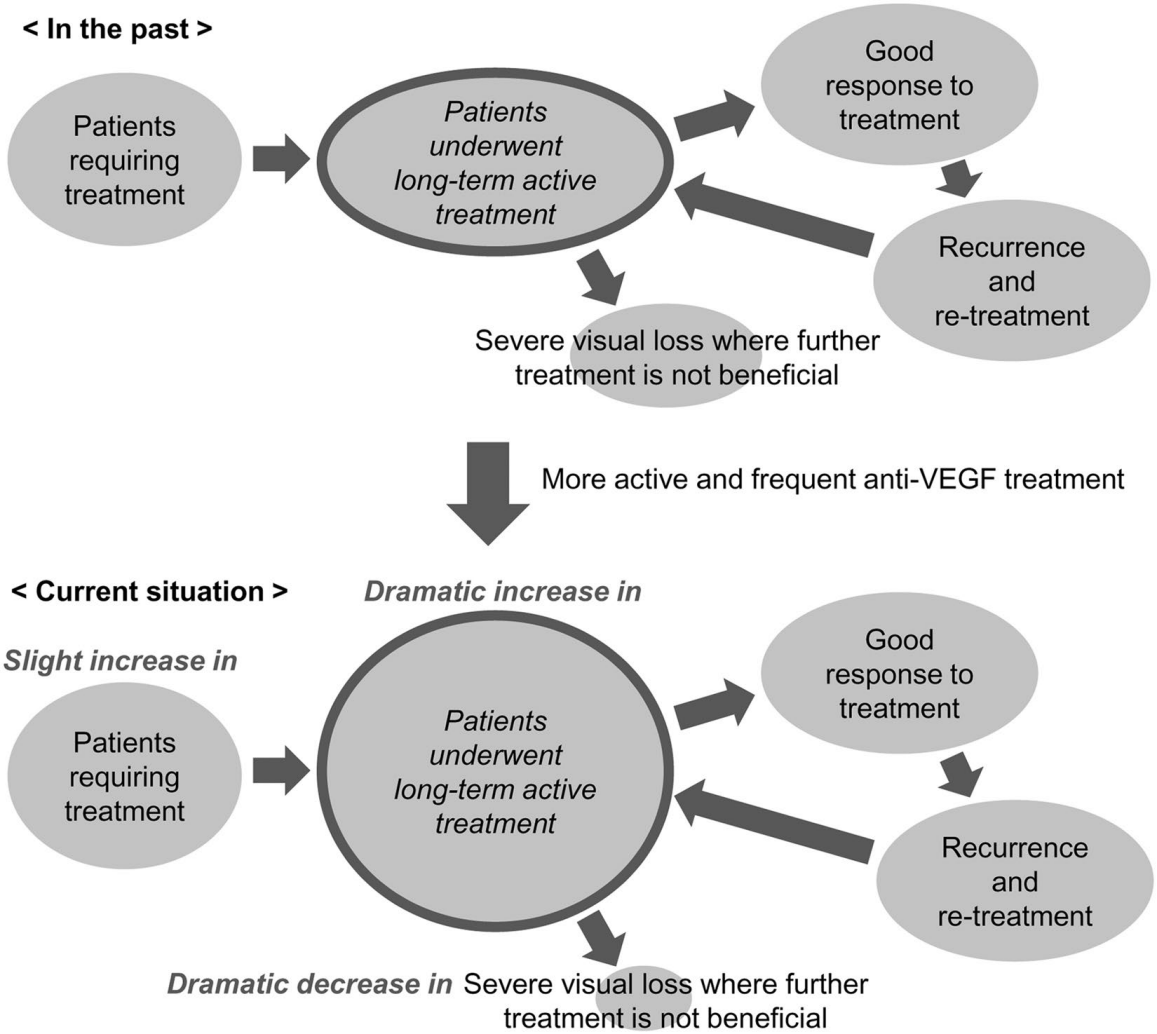
Schematic diagram of a postulated mechanism involved in the widespread administration of active and frequent anti-VEGF treatment leading to increase in the population of patients who underwent long-term active treatment.

Considering the results of the previous studies, we carefully postulate that active and frequent anti-VEGF injections along with the adaptation of a proactive regimen may lead to better treatment outcomes compared to the previous outcomes. The similar hypothesis was previously suggested by Cohen *et al*.^24^. As a result, some patients whose vision might have already been deteriorated because of previous undertreatment may have benefited from this new trend and have maintained their vision in the long-term. Sloan and Hanrahan previously showed the marked decrease in the incidence of visual loss after the introduction of anti-VEGF, suggesting that the technological advances led to important improvements in visual health^25^. We believe that a similar phenomenon occurs in our patients. In this case, it is not caused by the development of a new drug but by changing the method for using a drug. Eventually, the population of patients who underwent long-term active anti-VEGF treatment without experiencing blindness markedly increased in our study population despite the fact that there is only a slight increase in the number of newly diagnosed neovascular AMD cases over time. Figure 4 shows our hypothesis as a schematic diagram.

Previously, Korobelnik *et al*. estimated the number of eyes with treatable neovascular AMD yearly^4^. The incidence of new cases, treatment duration, probability of neovascularization in the fellow eye, death rate with advancing age, and population aging were taken into account to predict the number of eyes with treatable neovascular AMD. As a result, a marked increase in the number of eyes with treatable neovascular AMD over time was predicted. Additionally, the treatment duration was the most sensitive parameter, which is associated with this trend. Based on this result, the authors suggested that “new drugs will have an impact on treatment duration, and this should be anticipated by public health decision makers^4^”. The study by Korobelnik *et al*. was performed based on the data before anti-VEGF agent was popularly administered. We believe that the results of the present study, which was performed based on the data in the era of anti-VEGF treatment, may provide some evidence that the hypothesis of Korobelnik *et al*. is valid.

### Will this trend continue in the future

One important question is as follows: Will this trend be temporary, or will it continue in the future? When treating neovascular AMD with anti-VEGF, using the same method that was used in the initial clinical trials will be the most effective way. For ranibizumab, it is monthly fixed dosing^5^, and for aflibercept, it is bimonthly fixed dosing^26^. However, it is often difficult to apply these methods to clinical practice because of the economic and time constraints encountered when performing these procedures.

Fortunately, the patents for ranibizumab and aflibercept will expire within several years^27^ leading to the development of biosimilars. Although there have been some concerns regarding the biosimilars, one advantage is that they can significantly reduce the cost of the drugs^28^. For this reason, it is possible that the introduction of the biosimilars of ranibizumab and aflibercept will influence the practice pattern. More specifically, it may help to focus on more effective treatment rather than on efficient treatment.

One important focus of future drug development for neovascular AMD is development of a long-lasting drug that can reduce the number of hospital visits while providing appropriate treatment efficacy. A recent clinical trial on brolucizumab demonstrated that a 12-week treatment cycle for this new anti-VEGF drug may be viable in a relevant proportion of eyes^29^. Furthermore, experimental studies have suggested the possibility of introducing longer-lasting anti-VEGF drugs in the future^30,31^.

We believe that these two changes, reducing the cost of the drug and developing a longer-lasting drug, may lead to an increased patient adherence to treatment and improvement in treatment outcomes. Eventually, it may lead the increase in the number of patients who underwent long-term active treatment without experiencing blindness.

### How will this trend be taken into account when establishing future strategies for the treatment of neovascular AMD

#### Economic impact

The cost of expensive drugs and frequent visits to hospitals can greatly increase the economic burden of treatment for neovascular AMD. If the patient is continuously treated without experiencing blindness, it may reduce social costs due to blindness. Conversely, however, the cost of treatment itself may markedly increase in this situation. To date, the predicted future incidence has been generally considered to estimate the future treatment burden of neovascular AMD^2,32^. However, since there must be a difference in the treatment burden between patients who underwent active treatment and who did not, the incidence of disease alone may not accurately estimate the future treatment burden. We postulate that the increase in the number of patients receiving long-term active treatment may significantly contribute to the increasing treatment burden of neovascular AMD in the future.

#### Need for patient care strategy

To date, investigators usually have focused on the following question: “How are we going to treat the patients with AMD?” Although neovascular AMD is a refractory disease that requires long-term treatment, the issue on “how to care the patient?” has not received much attention. Patients with neovascular AMD may experience psychological depression and anxiety because of various reasons, including functional limitations due to decreased visual acuity, fear of injections, and fear of blindness^33,34^. However, psychiatric management of AMD patients has not been systematically implemented. Neovascular AMD usually develops in the elderly population^8,35^. Thus, the role of caregivers is very important for successful long-term treatment. However, treatment of neovascular AMD is associated with a considerable burden to caregivers^36,37^, with most caregivers experiencing distress even though they are the family members of the patients^37^. Therefore, to maintain long-term patient adherence to treatment, continuing education and care for the patients’ family and psychological care for patients will be necessary. With the growing population of patients undergoing long-term active treatment, the importance of this approach is expected to increase in the future.

In South Korea, reimbursement of the fee for ranibizumab and aflibercept treatment is not allowed for end-stage neovascular AMD by the national insurance system. For this reason, patients whose eyes show large fibrotic scars or diffuse geographic atrophy at initial presentation are usually treated with bevacizumab. In the bevacizumab group, the interval between the diagnosis of neovascular AMD and the last anti-VEGF injection has increased over time. However, unlike eyes initially treated with ranibizumab or aflibercept, an increasing trend of the total number of injections was not definitely noted after the 1st quarter of 2017 in the bevacizumab group. We postulated that the reason for this difference is that the vision of patients in the bevacizumab group was already severely impaired leading to discontinuing of the treatment after several injections due to a lack of treatment benefit.

The strength of the present study is that we first focused on the increasing population of patients receiving long-term active treatment for neovascular AMD. However, there are limitations of the present study. This study was retrospective, and analysis was performed based on the data from a single institution. Additionally, all the included patients were Korean. The timing of introduction of anti-VEGF therapy varies from country to country, and the preferred drugs and treatment regimen may also be different. Thus, further researches will be needed to identify whether similar trends are also noted in other countries. Lastly, patients who were initially treated with bevacizumab were separately analyzed. Hence, the continuous increase in the number of anti-VEGF injections over time which was one of the main results of the study, may not be valid for end-stage neovascular AMD cases.

In summary, results of the present study show that the population of patients who underwent long-term active anti-VEGF treatment for neovascular AMD markedly increased over time. As a result, these patients have mainly accounted for a dramatic increase in the number of anti-VEGF injections over time. We suggest that this trend should be taken into account in establishing future strategies for the treatment of neovascular AMD.

## References

[1] Flaxman SR (2017). Global causes of blindness and distance vision impairment 1990–2020: a systematic review and meta-analysis. The Lancet. Global health.

[2] Wong WL (2014). Global prevalence of age-related macular degeneration and disease burden projection for 2020 and 2040: a systematic review and meta-analysis. The Lancet. Global health.

[3] Colijn JM (2017). Prevalence of Age-Related Macular Degeneration in Europe: The Past and the Future. Ophthalmology.

[4] Korobelnik JF, Moore N, Blin P, Dharmani C, Berdeaux G (2006). Estimating the yearly number of eyes with treatable neovascular age-related macular degeneration using a direct standardization method and a markov model. Investigative ophthalmology & visual science.

[5] Rosenfeld PJ (2006). Ranibizumab for neovascular age-related macular degeneration. The New England journal of medicine.

[6] Day S, Acquah K, Lee PP, Mruthyunjaya P, Sloan FA (2011). Medicare costs for neovascular age-related macular degeneration, 1994–2007. American journal of ophthalmology.

[7] Prenner JL (2015). Disease Burden in the Treatment of Age-Related Macular Degeneration: Findings From a Time-and-Motion Study. American journal of ophthalmology.

[8] Park SJ, Kwon KE, Choi NK, Park KH, Woo SJ (2015). Prevalence and Incidence of Exudative Age-Related Macular Degeneration in South Korea: A Nationwide Population-Based Study. Ophthalmology.

[9] Rim, T. H., Yoo, T. K., Kim, S. H., Kim, D. W. & Kim, S. S. Incidence of exudative age-related macular degeneration and treatment load under the Korean national health insurance system in 2010–2015. *The British journal of ophthalmology* (2018). 10.1136/bjophthalmol-2018-312693 30573498

[10] Vaze A, Fraser-Bell S, Gillies M (2014). Reasons for discontinuation of intravitreal vascular endothelial growth factor inhibitors in neovascular age-related macular degeneration. Retina (Philadelphia, Pa.).

[11] Kruger Falk M, Kemp H, Sorensen TL (2013). Four-year treatment results of neovascular age-related macular degeneration with ranibizumab and causes for discontinuation of treatment. American journal of ophthalmology.

[12] Schmidt-Erfurth U (2014). Intravitreal aflibercept injection for neovascular age-related macular degeneration: ninety-six-week results of the VIEW studies. Ophthalmology.

[13] Fung AE (2007). An optical coherence tomography-guided, variable dosing regimen with intravitreal ranibizumab (Lucentis) for neovascular age-related macular degeneration. American journal of ophthalmology.

[14] Spaide R (2007). Ranibizumab according to need: a treatment for age-related macular degeneration. American journal of ophthalmology.

[15] Freund KB (2015). Treat-And-Extend Regimens with Anti-Vegf Agents in Retinal Diseases: a Literature Review and Consensus Recommendations. Retina (Philadelphia, Pa.).

[16] Group. WCFTUA-RMDEU (2014). The neovascular age-related macular degeneration database: multicenter study of 92 976 ranibizumab injections: report 1: visual acuity. Ophthalmology.

[17] Holz FG (2015). Multi-country real-life experience of anti-vascular endothelial growth factor therapy for wet age-related macular degeneration. The British journal of ophthalmology.

[18] Holz FG (2016). Key drivers of visual acuity gains in neovascular age-related macular degeneration in real life: findings from the AURA study. The British journal of ophthalmology.

[19] Wykoff CC (2015). Prospective Trial of Treat-and-Extend versus Monthly Dosing for Neovascular Age-Related Macular Degeneration: TREX-AMD 1-Year Results. Ophthalmology.

[20] Hatz K, Prunte C (2017). Treat and Extend versus Pro Re Nata regimens of ranibizumab in neovascular age-related macular degeneration: a comparative 12 Month study. Acta ophthalmologica.

[21] Rufai SR (2017). A systematic review to assess the ‘treat-and-extend’ dosing regimen for neovascular age-related macular degeneration using ranibizumab. Eye (London, England).

[22] Hatz K, Prunte C (2016). Changing from a pro re nata treatment regimen to a treat and extend regimen with ranibizumab in neovascular age-related macular degeneration. The British journal of ophthalmology.

[23] Kvannli L, Krohn J (2017). Switching from pro re nata to treat-and-extend regimen improves visual acuity in patients with neovascular age-related macular degeneration. Acta ophthalmologica.

[24] Cohen SY (2013). Ranibizumab for exudative AMD in a clinical setting: differences between 2007 and 2010. Graefe’s archive for clinical and experimental ophthalmology = Albrecht von Graefes Archiv fur klinische und experimentelle Ophthalmologie.

[25] Sloan FA, Hanrahan BW (2014). The effects of technological advances on outcomes for elderly persons with exudative age-related macular degeneration. JAMA ophthalmology.

[26] Heier JS (2012). Intravitreal aflibercept (VEGF trap-eye) in wet age-related macular degeneration. Ophthalmology.

[27] Sharma A, Reddy P, Kuppermann BD, Bandello F, Lowenstein A (2018). Biosimilars in ophthalmology: “Is there a big change on the horizon?”. Clinical ophthalmology (Auckland, N.Z.).

[28] Lyman GH, Zon R, Harvey RD, Schilsky RL (2018). Rationale, Opportunities, and Reality of Biosimilar Medications. The New England journal of medicine.

[29] Dugel PU (2017). Brolucizumab Versus Aflibercept in Participants with Neovascular Age-Related Macular Degeneration: A Randomized Trial. Ophthalmology.

[30] Lance KD (2016). *In vivo* and *in vitro* sustained release of ranibizumab from a nanoporous thin-film device. Drug delivery and translational research.

[31] Zhang L (2015). Coaxial Electrospray of Ranibizumab-Loaded Microparticles for Sustained Release of Anti-VEGF Therapies. PloS one.

[32] Saxena N, George PP, Hoon HB, Han LT, Onn YS (2016). Burden of Wet Age-Related Macular Degeneration and Its Economic Implications in Singapore in the Year 2030. Ophthalmic epidemiology.

[33] Dawson SR, Mallen CD, Gouldstone MB, Yarham R, Mansell G (2014). The prevalence of anxiety and depression in people with age-related macular degeneration: a systematic review of observational study data. BMC ophthalmology.

[34] McCloud C, Lake S (2015). Understanding the patient’s lived experience of neovascular age-related macular degeneration: a qualitative study. Eye (London, England).

[35] Friedman DS (2004). Prevalence of age-related macular degeneration in the United States. Archives of ophthalmology (Chicago, Ill.: 1960).

[36] Gohil R (2015). Caregiver Burden in Patients Receiving Ranibizumab Therapy for Neovascular Age Related Macular Degeneration. PloS one.

[37] Vukicevic M, Heraghty J, Cummins R, Gopinath B, Mitchell P (2016). Caregiver perceptions about the impact of caring for patients with wet age-related macular degeneration. Eye (London, England).

