# Author Correction: Increase in the Population of Patients with Neovascular Age-Related Macular Degeneration Who Underwent Long-Term Active Treatment

**DOI:** 10.1038/s41598-020-57960-5

**Published:** 2020-01-17

**Authors:** Seung Kook Baek, Jae Hui Kim, Jong Woo Kim, Chul Gu Kim

**Affiliations:** 1 Department of Ophthalmology, Konyang University College of Medicine, Daejeon, South Korea; 2 Department of Ophthalmology, Kim’s Eye Hospital, Konyang University College of Medicine, Seoul, South Korea

Correction to: *Scientific Reports* 10.1038/s41598-019-49749-y, published online 13 September 2019

The original version of this Article contained an error in the Abstract.

“This retrospective, observational study included 18,165 patients who received anti-vascular endothelial growth factor injections (3,974 eyes).”

now reads:

“This retrospective, observational study included 3,380 patients who received anti-vascular endothelial growth factor injections (3,974 eyes).”

This error has now been corrected in the PDF and HTML versions of the Article.

